# The Influence of Anti-ETAR and Anti-CXCR3 Antibody Levels on the Course of Specific Glomerulonephritis Types

**DOI:** 10.3390/jcm13247752

**Published:** 2024-12-19

**Authors:** Maciej Szymczak, Harald Heidecke, Marcelina Żabińska, Łucja Janek, Jakub Wronowicz, Krzysztof Kujawa, Karolina Bukowiec-Marek, Tomasz Gołębiowski, Karolina Skalec, Kai Schulze-Forster, Andrzej Konieczny, Mirosław Banasik

**Affiliations:** 1 Clinical Department of Nephrology, Transplantation Medicine and Internal Diseases, Wroclaw Medical University, 50-556 Wroclaw, Poland; karolina.bukowiec-marek@umw.edu.pl (K.B.-M.); tomasz.golebiowski@umw.edu.pl (T.G.); andrzej.konieczny@umw.edu.pl (A.K.); miroslaw.banasik@umw.edu.pl (M.B.); 2 CellTrendGmbh, Im Biotechnologiepark 3 TGZ II, 14943 Luckenwalde, Germany; heidecke@celltrend.de (H.H.); schufo@celltrend.de (K.S.-F.); 3 Department of Preclinical Sciences, Pharmacology and Medical Diagnostics, Faculty of Medicine, Wrocław University of Science and Technology, 58-376 Wroclaw, Poland; marcelina.zabinska@pwr.edu.pl; 4 Statistical Analysis Center, Wroclaw Medical University, 50-368 Wroclaw, Poland; lucja.janek@umw.edu.pl (Ł.J.); jakub.wronowicz@umw.edu.pl (J.W.); krzysztof.kujawa@umw.edu.pl (K.K.); 5 Clinical Department of Nephrology, Transplantation Medicine and Internal Diseases, University Clinical Hospital, 50-556 Wroclaw, Poland; kskalec@usk.wroc.pl

**Keywords:** antibodies, endothelin A receptor, CXC motif chemokine receptor 3, glomerulonephritis

## Abstract

**Background:** Anti-ETAR (endothelin A receptor) antibodies and anti-CXCR3 (C-X-C motif chemokine receptor 3) antibodies are types of non-HLA (human leukocyte antigens) antibodies that could have some influence on the course of glomerulonephritis. The authors aimed to study the influence of these antibodies’ levels on the course of specific glomerulonephritis types. **Methods:** We evaluated the anti-ETAR and anti-CXCR3 antibody levels in the serum of patients with membranous nephropathy (n = 18), focal and segmental glomerulosclerosis (FSGS) (n = 25), systemic lupus erythematosus (n = 17), IgA nephropathy (n = 14), mesangiocapillary glomerulonephritis (n = 6), anti-neutrophil cytoplasmic antibodies (c-ANCA) vasculitis (n = 40), and perinuclear anti-neutrophil cytoplasmic antibodies (p-ANCA) vasculitis (n = 16), and we compared their levels with the control group (n = 22). Next, we observed the patients’ clinical parameters (serum creatinine, albumin, total protein) for 2 years and checked the correlation of the clinical course markers with basic receptor antibody level. **Results:** Our results indicate lower anti-ETAR antibody levels in patients with FSGS and IgA nephropathy compared to the control group. Both types of antibodies correlated with creatinine levels after 2 years of observation in IgA nephropathy. Both types of antibodies seemed to negatively influence the total protein and albumin levels in systemic lupus erythematosus. **Conclusions**: This prospective observation showed that anti-ETAR and anti-CXCR 3 antibody levels are connected with the course of IgA nephropathy and lupus nephritis.

## 1. Introduction

Glomerular diseases are autoimmunological disorders that involve many autoantibodies, and predicting the disease course is challenging. Furthermore, appropriate glomerular disease progression markers are still lacking.

Many common autoantibodies associate with specific glomerular diseases, including anti-PLA2R (phospholipase A2 receptor) antibodies (membranous nephropathy) [1], ANA (antinuclear antibodies) (lupus nephritis) [2], c-ANCA (cytoplasmic-anti-neutrophil cytoplasmic antibodies) (granulomatosis with polyangiitis = c-ANCA vasculitis) [3], and p-ANCA (perinuclear–cytoplasmic anti-neutrophil antibodies) (microscopic vasculitis = p-ANCA vasculitis) [4]. In addition, diseases such as lupus nephritis are concomitant with numerous other autoantibodies [5].

Despite patients receiving immunosuppression, transplanted kidneys remain a source of many antigens that are capable of immunological activation, with HLAs (human leukocyte antigens) proving the most important [6]. Antibodies against HLAs are directly connected with humoral rejection activation, though many other antigens relate to graft function. Moreover, some antibodies are linked to components of kidney transplantation rejection, including arteritis [7]. 

Non-HLA antigens [8] include autoantibodies against AT1R (angiotensin II type 1 receptor) [9], AT2R (angiotensin II type 2 receptor) [10], and ETAR (endothelin A receptor) [11]. Other lesser-known antigens may be classified into these groups, such as CXCR3 (C-X-C chemokine receptor 3) autoantibodies [12].

Studies suggest that non-HLA antibodies, especially anti-ETAR, may exist in patients before kidney transplantation [13], which has led to the supposition that glomerulonephritis and autoimmunological disease patients are more prone to these antibodies and the subsequent decision to determine their levels in glomerulonephritis patients. Furthermore, anti-ETAR and anti-CXCR3 antibodies are found in healthy individuals, increasing the likelihood that they are active in glomerulonephritis patients and that the evaluation would produce crucial results. Moreover, earlier findings that anti-CXCR3 antibodies may be a marker of cardiovascular disease [12] increase their clinical importance and future opportunities to develop drugs targeting them.

ETAR is involved in the development of glomerular inflammation, peritubular fibrosis, and cell proliferation control [14], with the receptor expression being linked with some kidney diseases, including ischemia–reperfusion injury (the activation of ETAR ameliorates this injury) [15], acute tubular necrosis and antibody-mediated rejection of transplanted kidneys [16], FSGS (focal segmental glomerulosclerosis) (nephrin loss and increased oxidative stress marker levels during the disease course) [17], mesangial proliferative nephritis [18], scleroderma renal crisis [19], and IgA (immunoglobulin A) nephropathy (a blockade of ETAR reveals a renoprotective effect and diminishes proteinuria in IgA nephropathy patients) [20].

Higher ETAR antibody concentrations are a risk factor for worse kidney function 12 months after transplantation [13], and patients with active c-ANCA vasculitis have higher ETAR antibody levels than those in remission [21]. These antibodies are involved in arteritis development and correlate with IL (interleukin)-8 in children after kidney transplantation [7].

ETAR antibodies are agonistic [11,22] and bind with the receptor for a prolonged time, amplifying the effects of ETAR activation [23]. Furthermore, the antibodies interact with AT1R antibodies to enhance and prolong immunological responses and inflammation [22].

Many cell types express CXCR3 receptors, especially T lymphocytes [24], and they are involved in inflammation development through the regulation of T lymphocyte migration to sites of inflammation and their differentiation into active forms [25]. The activation of CXCR3 also directs macrophages to sites of inflammation [26] and functions in angiogenesis regulation [27].

Certain kidney diseases involve CXCR3 in their development, with glucocorticoids having been shown to diminish CXCR3+ T cell influx during c-ANCA vasculitis and cause increased CXCR3+ T cell numbers in kidney biopsies from p-ANCA vasculitis patients, indicating a poorer prognosis [28]. High CXCR3 expressions are found in the mesangium of kidney biopsies taken from active cases of lupus nephritis [29], IgA nephropathy [30], membranoproliferative glomerulonephritis, and rapidly progressive glomerulonephritis [31]. Furthermore, CXCR3 is implicated in the progressive loss of renal function [32] and renal ischemia–reperfusion injury [33]. 

CXCR3 antibodies impact Sjogren syndrome development, with lower anti-CXCR3 autoantibody levels being found in Sjogren syndrome patients than in healthy individuals [34]. Furthermore, anti-CXCR3 antibody levels predict the deterioration of lung function in systemic sclerosis patients [35], while anti-CXCR3 antibodies predict cardiovascular risk in patients with cardiovascular diseases [36].

CXCR3 antibodies are agonistic and activate the CXCR3 receptor [36]. 

As such, we assessed anti-ETAR and anti-CXCR3 antibody levels in patients with specific forms of glomerulonephritis and compared them with those of healthy controls. Next, we planned to observe our patients’ clinical parameters for two years to determine if basic antibody levels influence the course of specific glomerular diseases.

## 2. Materials and Methods

We gathered serum from patients with specific kinds of glomerulonephritis and a healthy control group (total n = 158). Materials included those derived from patients with membranous nephropathy (n = 18), focal and segmental glomerulosclerosis (FSGS) (n = 25), lupus nephropathy (n = 17), IgA nephropathy (n = 14), proliferative mesangial non-IgA glomerulonephritis (n = 6), c-ANCA positive vasculitis (n = 40), and p-ANCA positive vasculitis (n = 16). The control group included 22 healthy Caucasian individuals without proteinuria, with a creatinine level no higher than 1.3 mg/dL, and without a history of kidney or autoimmunological diseases. All the patients and all the people in the control group came from Poland. Everybody who was included in the study signed an informed consent form before the collection of material. We obtained permission from The Bioethics Committee at Wroclaw Medical University to perform this study (No. KB-221/2023 and KB-546/2012). The groups of patients were numbered as follows: 1—patients with membranous nephropathy, 2—patients with FSGS, 3—patients with lupus nephropathy, 4—patients with IgA nephropathy, 5—patients with proliferative mesangial non-IgA glomerulonephritis, 6—control group, 7—patients with c-ANCA positive vasculitis, and 8—patients with p-ANCA positive vasculitis. We included patients in the study between 2013 and 2020. There were other patients who were treated in our clinic during this time who were not randomized into this study. 

All diagnoses were histopathologically confirmed. Only patients with a kidney biopsy result were recruited into the study. 

The patients arrived at our clinic because of the first signs and symptoms of the disease or relapse of the disease. We included them in the study before the start of intensive treatment (e.g., steroid pulses, cyclophosphamide).

Inclusion in this study required a result of histopathological kidney evaluation indicating one of the following diagnoses: membranous nephropathy, FSGS, lupus nephropathy, IgA nephropathy, proliferative mesangial non-IgA glomerulopathy, or c-ANCA or p-ANCA positive vasculitis. Moreover, only patients with proteinuria in the last accessible urine evaluation before the term of the material collection were included in the study.

Exclusion criteria included patients with a history of dialysis or kidney transplantation, past or present malignancy, and active infection at the moment of material collection.

**Figure 1:**
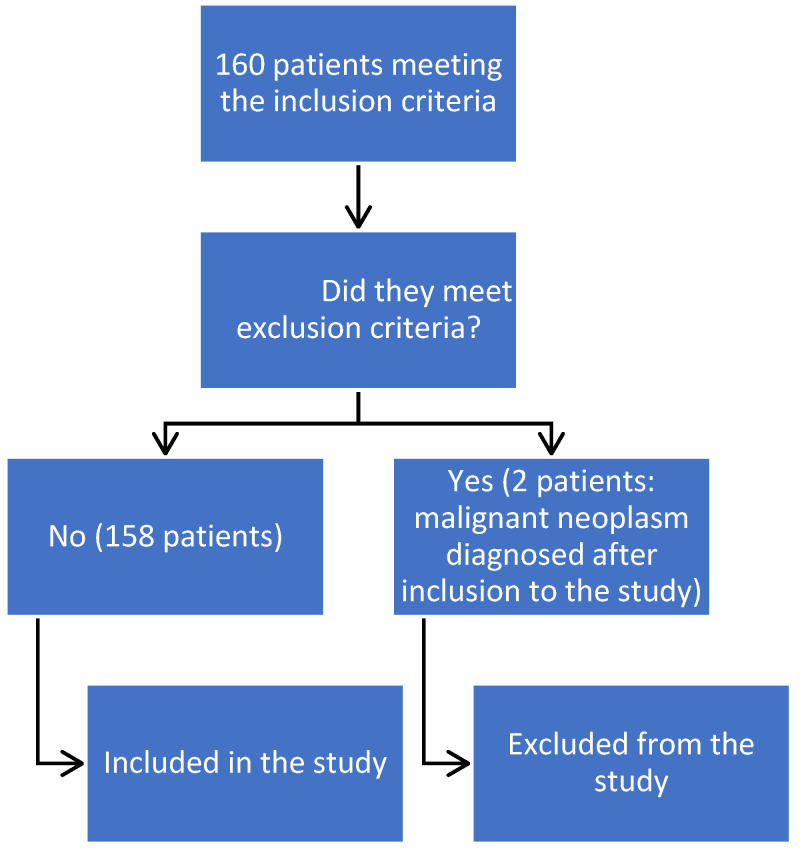
Flowchart presenting the process of including in the study.

A flowchart presenting the inclusion process in this study is presented in Figure 1.

Blood was collected from the patients simultaneously with blood collection for standard laboratory evaluation, without additional vein puncture. A volume of 2.7 mL of blood was collected. Patients were not obligated to fast the morning before the vein puncture. 

The laboratory was located very close to the clinical ward, so it was possible to transport the materials swiftly. We started centrifugation of all the samples exactly 10 min after material collection to ensure that there would be no bias associated with different material storage times before freezing. The time was checked. We did not collect material in cases where the temperature was > 28 °C. After that, we performed blood centrifugation at 1500 g for 10 min. Next, the obtained serum was frozen at −80 °C. 

The serum concentrations of ETAR and CXCR3 antibodies were evaluated with the usage of commercially available enzyme-linked immunosorbent assay (ELISA) kits (producer: CellTrend, Luckenwalde, Germany) following the manufacturer’s instructions. We evaluated the samples on a precoated microtiter plate. We diluted samples to a 1:100 concentration. Next, we added standards and samples into the wells and incubated them for 2 h at a temperature of 2–8 °C. After that, we performed the washing steps. Next, we detected non-human leukocyte antigen (HLA) antibodies with horseradish peroxidase (POD)-labeled anti-human IgG antibody (1:100), which was developed in color with a 3, 3′, 5, 5′-tetramethylbenzidine (TMB) substrate solution. Measurements were performed at 450 nm with a correction wavelength of 630 nm. We converted the optical density into the concentration with standard curve usage. A result of the test ≥ 2.5 U/mL was identified as a positive result. Levels < 2.5 U/mL were considered negative. These ranges refer to both kinds of antibodies. Antibody ELISA assays were validated. The validation process was performed in accordance with the Bioanalytical Method Validation from the Food and Drug Administration (FDA)’s Guidance for Industry. There were no significant cross-reactions with other lupus antibodies. 

We collected the following clinical data: basic serum creatinine, proteinuria, basic serum total protein, basic serum albumin, age, and sex of patients.

Creatinine, total protein, and albumin serum levels were also checked 1, 3, 6, and 12 months and 2 years after the basic evaluation. 

### Statistical Analysis

We compared ETAR and CXCR3 antibody concentrations, serum creatinine, serum albumin, serum total protein, proteinuria, and age between the specific glomerulonephritis groups and the control group using the Kruskal–Wallis test and Dunn’s test with Bonferroni correction or analysis of variance (ANOVA).

We performed a correlation analysis between the quantitative variables. We assessed the data distribution with the usage of the Shapiro–Wilk test. We used Pearson`s or Spearman`s correlation coefficient depending on the kind of distribution in the specimens (normal or non-normal). A T-test was used to check the significance of the correlations. We considered the results of analyses as positive when *p* < 0.05. The correlations between ETAR and CXCR3 antibody levels and clinical data were checked. We also performed an analysis of the correlations between the levels of antinuclear antibodies (ANAs) in lupus nephropathy patients, c-ANCA in granulomatosis with polyangiitis patients, p-ANCA in p-ANCA vasculitis patients, and ETAR and CXCR3 antibody concentrations. 

Next, we performed an analysis of the variability of the clinical factors (serum creatinine, serum albumin, and serum total protein levels) over 2 years of observations depending on basic ETAR and CXCR3 antibody levels with usage:

- Trend evaluation: Spearman’s correlation between ETAR and CXCR3 levels and time (months);
- Statistical range evaluation based on Spearman’s correlation;
- Standard deviation evaluation based on Spearman’s correlation;
- Coefficient of variation evaluation based on Spearman’s correlation.

We checked the correlations between ETAR and CXCR3 antibodies for all the groups. All the analyses were performed using STATISTICA 13.

## 3. Results

The clinical data of the patients are presented in Table 1. Values in red indicate statistical difference from the others.

The creatinine levels in p-ANCA vasculitis patients were higher than those of the control group (*p* < 0.05). Patients from the control group had higher total protein and albumin levels than the other groups (*p* < 0.05). Patients from the control group had no proteinuria. Other differences were not statistically significant.

**Figure 2:**
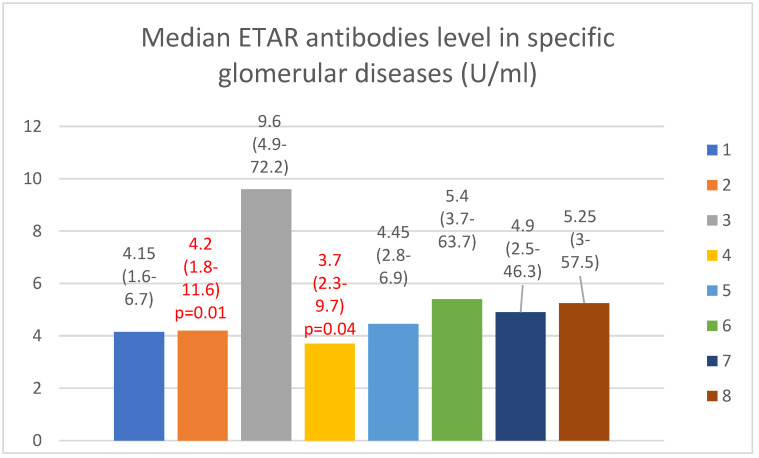
Median ETAR antibody levels in specific glomerular diseases (U/mL). Key: 1—membranous nephropathy; 2—focal and segmental glomerulosclerosis; 3—lupus nephritis; 4—IgA nephropathy; 5—mesangial proliferative (non-IgA) glomerulonephritis; 6—control group; 7—c-ANCA vasculitis; 8—p-ANCA vasculitis. The groups with values in red were statistically lower than the control group.

The anti-ETAR antibody serum median levels are presented in Figure 2.

**Figure 3:**
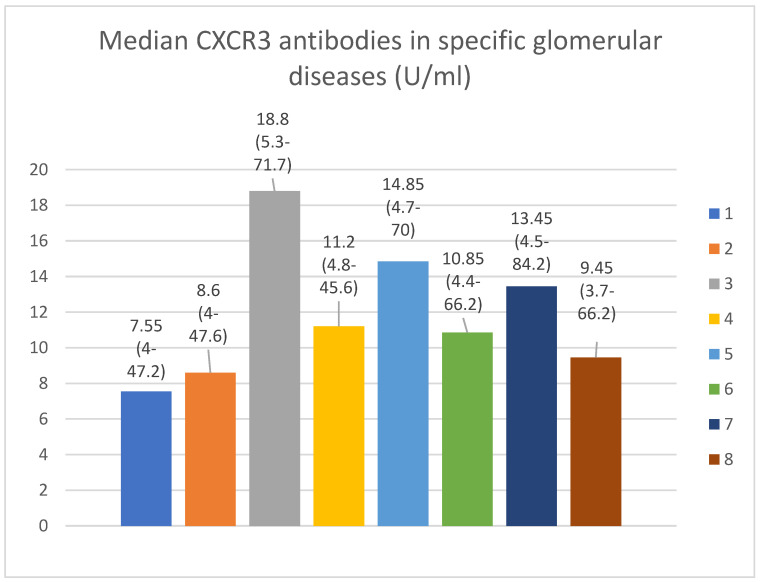
Median CXCR 3 antibodies in specific glomerular diseases (U/mL). Key: 1—membranous nephropathy; 2—focal and segmental glomerulosclerosis; 3—lupus nephritis; 4—IgA nephropathy; 5—mesangial proliferative (non-IgA) glomerulonephritis; 6—control group; 7—c-ANCA vasculitis; 8—p-ANCA vasculitis.

The anti-CXCR3 antibody serum median levels are presented in Figure 3. 

**Figure 4:**
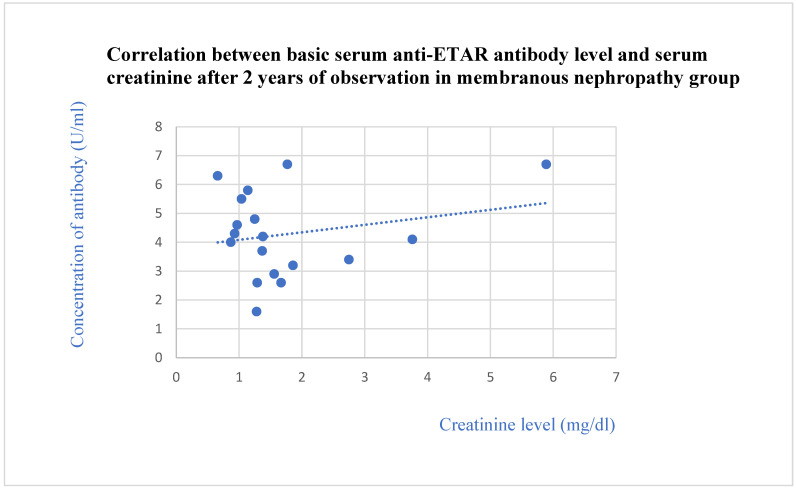
Correlation between basic serum anti-ETAR antibody level and serum creatinine after 2 years of observation in membranous nephropathy patients (r = 0.55; *p* = 0.03). Blue dots represent particular patients. Blue dotted line—trend line.

**Figure 5:**
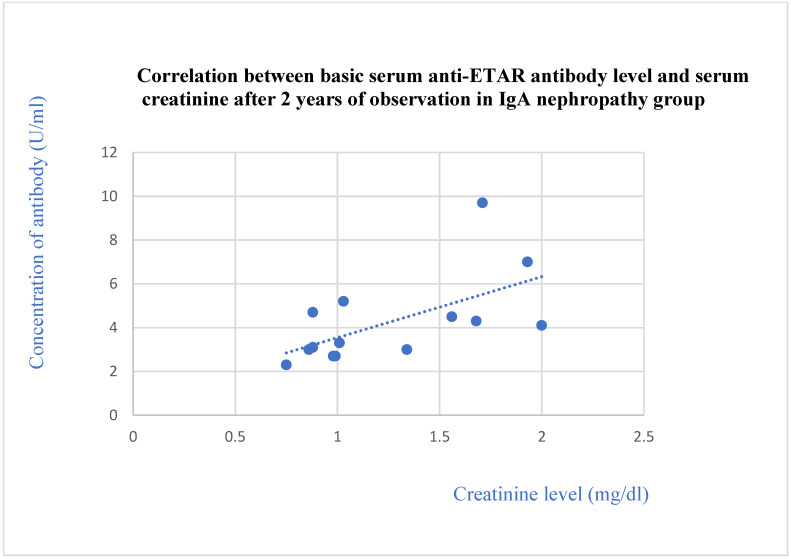
Correlation between basic serum anti-ETAR antibody level and serum creatinine after 2 years of observation in IgA nephropathy patients (r = 0.57; *p* = 0.03). Blue dots represent particular patients. Blue dotted line—trend line.

We found that the anti-ETAR antibody levels were lower in patients with FSGS and IgA nephropathy than in the control group (*p* = 0.01 and 0.04, respectively). The anti-ETAR antibody levels in lupus nephritis patients were higher than those of the membranous nephropathy (*p* = 0.0001), FSGS (*p* = 0.0001), IgA nephropathy (*p* = 0.0001), and c-ANCA vasculitis (*p* = 0.002) groups. The basic anti-ETAR levels in patients with membranous nephropathy (Figure 4) and IgA nephropathy (Figure 5) correlated positively with creatinine levels after 2 years of observation (*p* = 0.03 in both groups of patients). 

**Figure 6:**
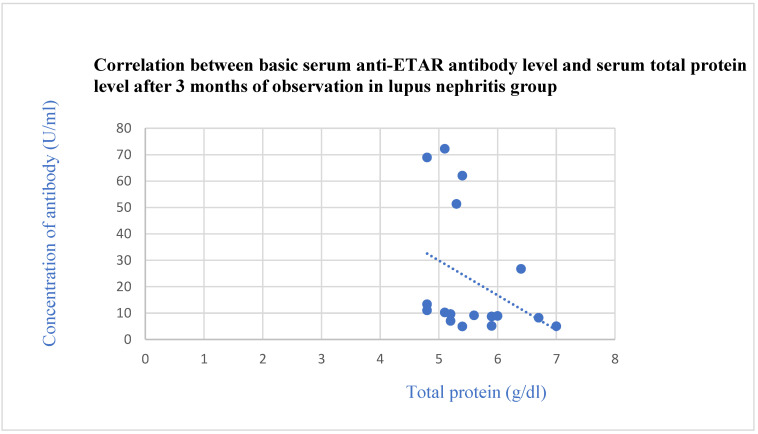
Correlation between basic serum anti-ETAR antibody level and serum total protein level after 3 months of observation in lupus nephritis group (r = −0.54; *p* = 0.03). Blue dots represent particular patients. Blue dotted line—trend line.

**Figure 7:**
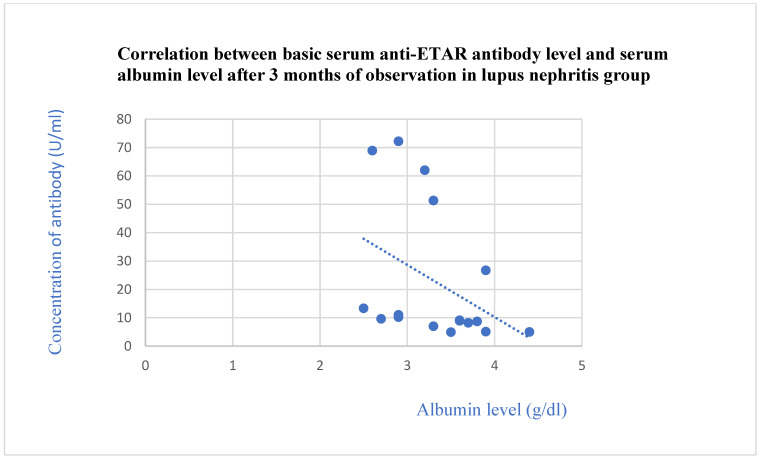
Correlation between basic serum anti-ETAR antibody level and serum albumin level after 3 months of observation in lupus nephritis group (r = −0.62; *p* = 0.01). Blue dots represent particular patients. Blue dotted line—trend line.

**Figure 8:**
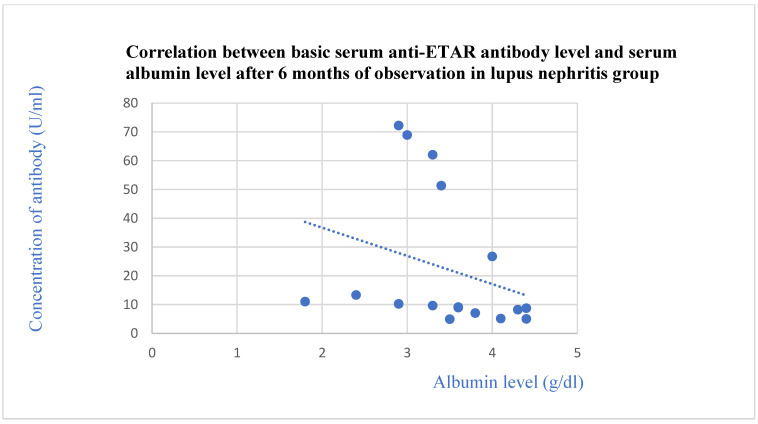
Correlation between basic serum anti-ETAR antibody level and serum albumin level after 6 months of observation in lupus nephritis group (r = −0.57; *p* = 0.03). Blue dots represent particular patients. Blue dotted line—trend line.

The basic anti-ETAR antibodies correlated negatively with the total protein level after 3 months (*p* = 0.03, Figure 6) and with the albumin level after 3 (*p* = 0.01, Figure 7) and 6 months (*p* = 0.03, Figure 8) of observation in the systemic lupus erythematosus group.

**Figure 9:**
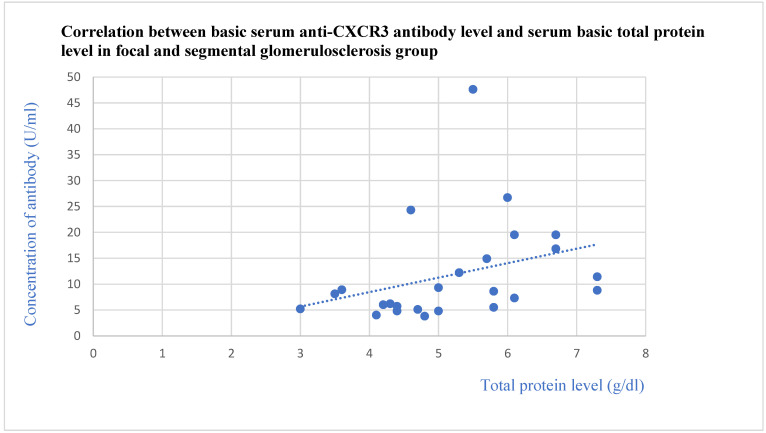
Correlation between basic serum anti-CXCR3 antibody level and basic serum total protein level in focal and segmental glomerulosclerosis group (r = 0.50; *p* = 0.009). Blue dots represent particular patients. Blue dotted line—trend line.

**Figure 10:**
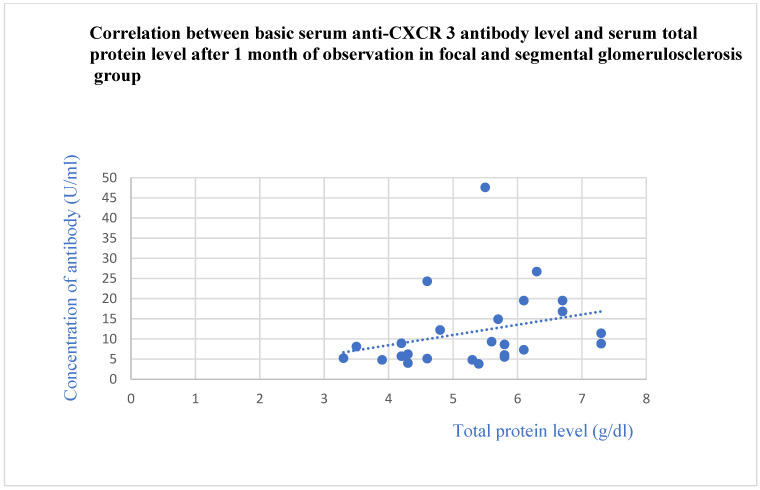
Correlation between basic serum anti-CXCR3 antibody level and serum total protein level after 1 month of observation in focal and segmental glomerulosclerosis group (r = 0.49; *p* = 0.02). Blue dots represent particular patients. Blue dotted line—trend line.

**Figure 11:**
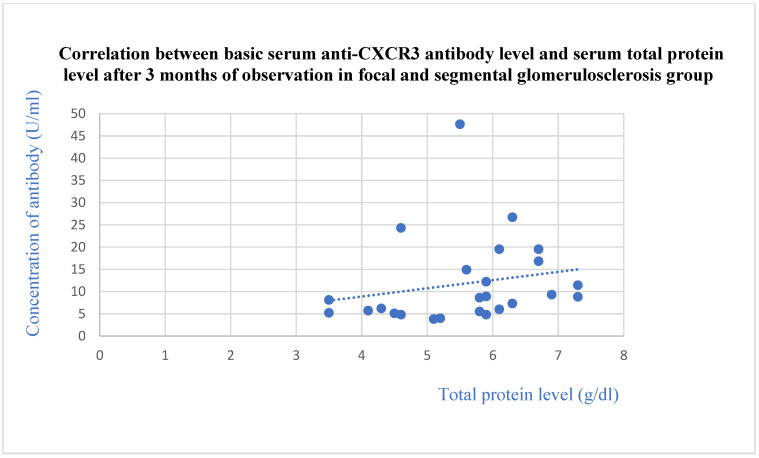
Correlation between basic serum anti-CXCR3 antibody level and serum total protein level after 3 months of observation in focal and segmental glomerulosclerosis group (r = 0.43; *p* = 0.04). Blue dots represent particular patients. Blue dotted line—trend line.

The basic anti-CXCR3 antibodies did not differ between the control group and specific glomerular diseases. The basic anti-CXCR3 antibody level correlated with the basic total protein levels (*p* = 0.009, Figure 9) and total protein level after 1 (*p* = 0.02, Figure 10) and 3 months (*p* = 0.04, Figure 11) of observation in the FSGS patients. 

**Figure 12:**
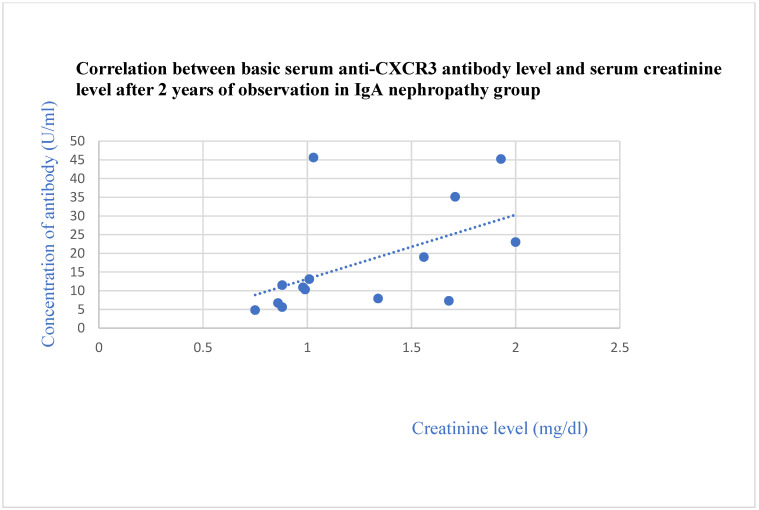
Correlation between basic serum anti-CXCR3 antibody level and serum creatinine level after 2 years of observation in IgA nephropathy group (r = 0.65; *p* = 0.01). Blue dots represent particular patients. Blue dotted line—trend line.

The basic anti-CXCR3 antibodies correlated with the creatinine level after 2 years in the IgA nephropathy group (*p* = 0.01, Figure 12). 

**Figure 13:**
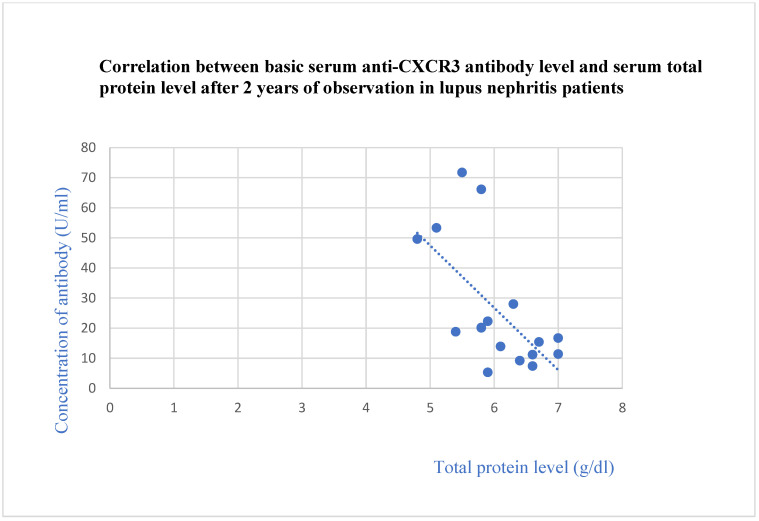
Correlation between basic serum anti-CXCR3 antibody level and serum total protein level after 2 years of observation in lupus nephritis group (r = −0.58; *p* = 0.03). Blue dots represent particular patients. Blue dotted line—trend line.

**Figure 14:**
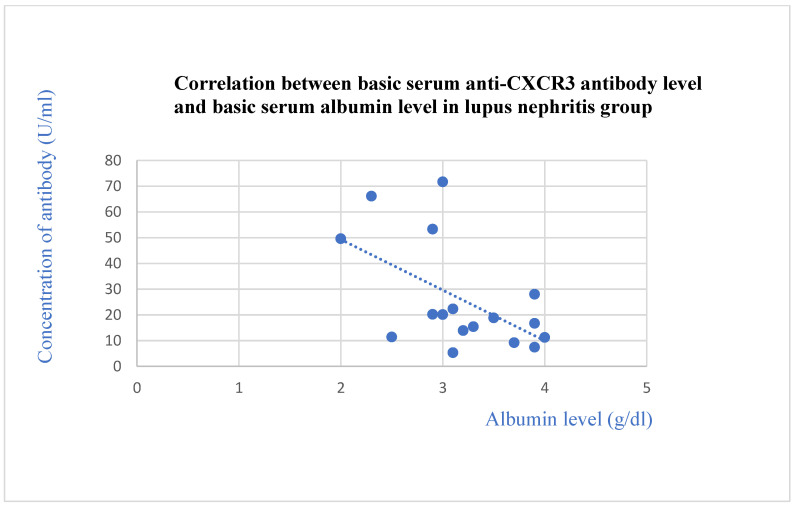
Correlation between basic serum anti-CXCR3 antibody level and basic serum albumin level in lupus nephritis group (r = −0.53; *p* = 0.02). Blue dots represent particular patients. Blue dotted line—trend line.

**Figure 15:**
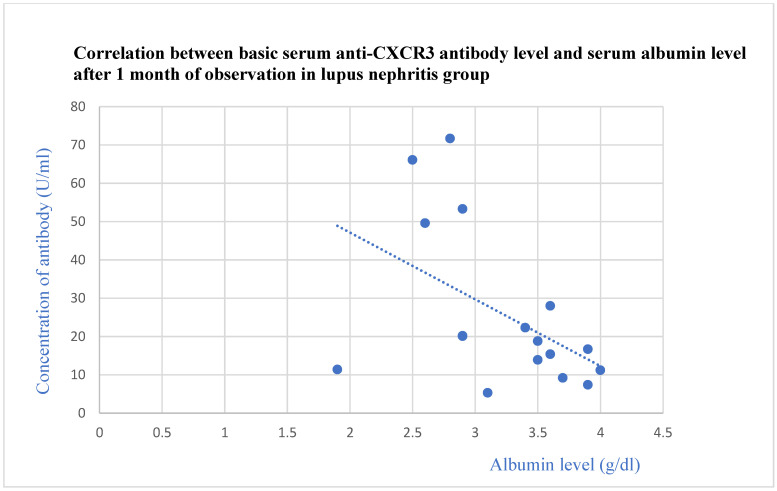
Correlation between basic serum anti-CXCR3 antibody level and albumin level after 1 month of observation in lupus nephritis group (r = −0.51; *p* = 0.04). Blue dots represent particular patients. Blue dotted line—trend line.

The anti-CXCR3 antibody level correlated negatively with the total protein level after 2 years of observation (*p* = 0.03, Figure 13) and with the basic albumin level (*p* = 0.02, Figure 14) and albumin level after 1 month of observation (*p* = 0.04, Figure 15) in the systemic lupus erythematosus group. 

**Figure 16:**
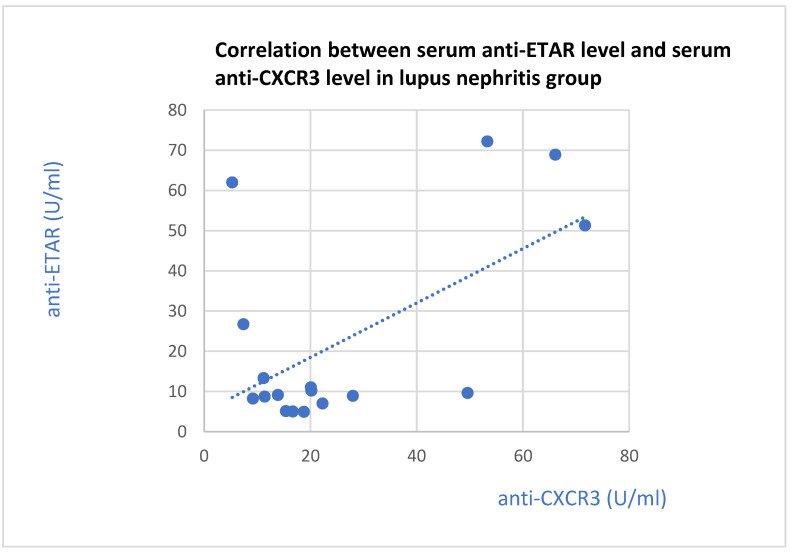
Correlation between basic serum ETAR antibody level and serum CXCR3 antibody level in lupus nephritis group (r = 0.58; *p* = 0.01). Blue dots represent particular patients. Blue dotted line—trend line.

**Figure 17:**
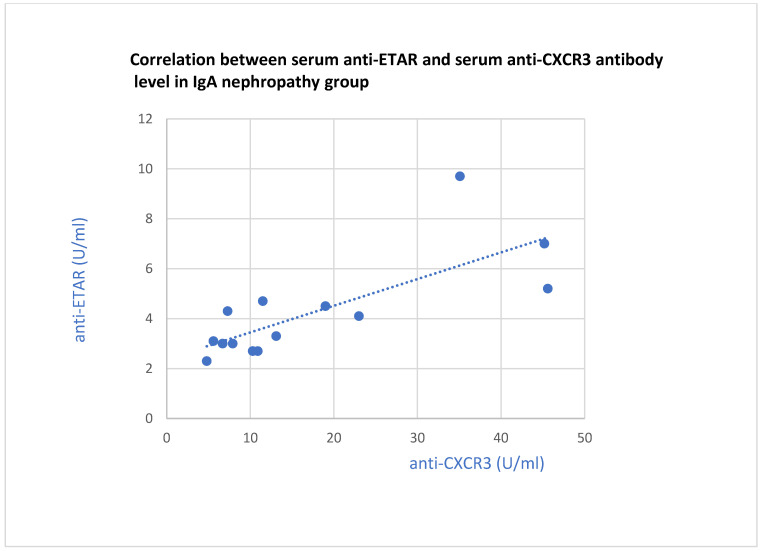
Correlation between basic serum ETAR antibody level and serum CXCR3 antibody level in IgA nephropathy group (r = 0.76; *p* = 0.001). Blue dots represent particular patients. Blue dotted line—trend line.

The anti-ETAR antibody correlated with the anti-CXCR3 antibody in the groups of patients with lupus nephritis (*p* = 0.01, Figure 16) and IgA nephropathy (*p* = 0.001, Figure 17).

Values of anti-ETAR antibodies concentrations in specific groups of patients are presented in Figure S1 and anti-CXCR3 antibodies in Figure S2.

## 4. Discussion

We found that the anti-ETAR antibody levels were lower in the IgA nephropathy and FSGS groups than in the control group.

The median anti-ETAR level in our healthy control group was lower than the median anti-ETAR level in the healthy control group from another study that used the same test (5.4 vs. 10.2 IU/mL) [37]. This difference likely reflects the relatively small size of both control groups (although our group was bigger: n = 22 vs. 12) and the relatively wide range in our group. Despite that fact, our IgA nephropathy and FSGS patients still had significantly lower anti-ETAR levels than our healthy control group.

The lower levels of ETAR antibody in the IgA nephropathy group may be surprising, but at the same time, their higher levels may be connected according to our results for patients with a worse prognosis.

A previous study [20] indicated that the blockade of ETAR diminishes proteinuria in patients with IgA nephropathy and causes renoprotection. The evaluated ETAR antibody is supposed to have an activating influence on the ETAR [22]. High ETAR antibody levels may reveal high activity of the disease.

ETAR was found to increase levels of the oxidative stress marker 8-oxo-guanine via the staining of biopsies from patients with FSGS and was connected with podocyte injury (nephrin loss) in these patients [17]. This observation suggests a potential connection between anti-ETAR and the processes connected with the pathogenesis of FSGS. A low level of anti-ETAR antibodies in serum may suggest that their consumption is connected with the binding of antigen in tissues [11] or regulatory function. The regulatory function of these antibodies in preeclampsia was suggested in another study [38]. For FSGS, this is a hypothesis and should be checked using molecular biology models.

Moreover, we found that anti-ETAR antibodies were higher in lupus nephritis patients compared to membranous nephropathy, FSGS, and c-ANCA vasculitis patients. Such results are expected, considering that lupus is a very active immunological disease and connected with the appearance of many autoantibodies [39]. The mean anti-ETAR antibody level in the lupus nephritis group, 22.48 ± 24.35 U/mL, was 2-fold than that of the control group (11.7 ± 15.47 U/mL). It is probable that in the case of bigger groups of patients, this difference would be statistically important. 

We observed a correlation between the basic level of ETAR antibodies and creatinine level after 2 years of observation in membranous nephropathy. There is no publication about ETAR or ETAR antibodies and membranous nephropathy. Nevertheless, membranous nephropathy is an autoimmunologic disease with the common presence of anti-PLA2R antibodies and other antibodies in serum [1,40]. The appearance of the systemic antibodies in the course of membranous nephropathy increases the probability that ETAR antibodies also have some connection with the pathogenesis of the disease, or that they are markers of the disease. Our results suggest that a higher ETAR antibody level may be a negative predictor of the prognosis in membranous nephropathy, but this requires confirmation based on bigger groups of patients. The ETAR antibodies in our group did not correlate with the PLA2R antibody level, so the real mechanism of ETAR antibodies’ connection with the pathogenesis of membranous nephropathy is still unknown.

The median level of anti-CXCR3 antibodies in our healthy control group was 10.85 IU/mL. This was very similar to the median healthy control group level in another study that used the same test for anti-CXCR3 antibody detection (11.1 IU/mL) [34].

The results of the anti-CXCR3 antibody evaluation and correlation with clinical parameters of the patients suggest a negative influence of higher antibody levels with the course of IgA nephropathy and lupus nephritis. In the case of lupus nephritis, this phenomenon may reflect the activity of lupus and the general tendency to indicate autoantibodies. There are some data indicating a connection of anti-CXCR3 antibodies with inflammation and suggesting the agonistic influence of these antibodies on CXCR3 [36]. Moreover, there are data suggesting an inverse correlation between the glomerular CXCR3 expression in kidneys from patients with lupus nephritis and proteinuria in these group of patients. The tuberointerstitial expression of CXCR3 was negatively correlated with the creatinine of the patients in this study [41]. Our data, in combination with accessible data, suggest a negative influence of anti-CXCR3 antibodies on the course of lupus nephritis.

The results for IgA nephropathy are more difficult to interpret. We observed a correlation between anti-CXCR3 antibody and creatinine levels after 2 years of observation. Simultaneously, the anti-CXCR3 antibody level correlates positively with the total basic protein level after 1 and 3 months of observations. There is, of course, a high probability that patients who had a high total basic protein level will still have a high total protein level in short observations, especially compared to patients who had a low total basic protein level. On the other hand, some factors may positively influence the total protein levels in glomerulonephritis patients [42] and negatively influence the creatinine level. Such factors are typically drugs like angiotensin-converting enzyme (ACE) inhibitors and cyclosporin [43].

After basic material collection before the start of the treatment, our patients were treated. ACE inhibitors are the standard treatment for patients with proteinuria and glomerulonephritis.

Nevertheless, the influence of anti-CXCR3 antibodies on the course of FSGS and IgA nephropathy cannot be excluded and should be checked using bigger groups of patients who are divided into subgroups according to the dosage of ACE inhibitor received.

During the course of observation, our patients received immunosuppressive treatment consisting of three doses of 500 mg of methylprednisolone, followed by oral prednisone starting at 1 mg/kg of body weight and subsequently diminished. This was the therapeutic scheme for patients with membranous glomerulonephritis, FSGS, and mesangial proliferative glomerulonephritis without IgA deposits. Patients with IgA nephropathy were treated with methylprednisolone at 500 mg every 2 months (three times). They also received prednisone at 0.5 mg per kg of body weight every other day for 6 months. Lupus nephritis and vasculitis patients received 500 mg of cyclophosphamide every 2 weeks (six times). Moreover, they received prednisone, initially at 1 mg/kg of body weight and then tapered down, and azathioprine at 100 mg/day.

The anti-CXCR3 antibodies that we detected are agonistic to CXCR3. Such a type of antibody exists naturally in healthy persons and people with different diseases [36]. Antagonistic anti-CXCR 3 antibodies (CXCR3-173) are also described in the literature, but this kind of antibody is a laboratory product that is used to test molecular models [44]. 

The positive correlation between anti-ETAR and anti-CXCR3 antibodies in the serum of lupus nephritis and IgA nephropathy patients may suggest systemic activation in these diseases. Systemic lupus erythematosus is a systemic disease. IgA nephropathy may also exhibit systemic signs in the course of Henoch–Schonlein syndrome [45]. A joint influence of the antibodies on the course of the diseases is also possible, and simultaneous analyses of many antibodies may be useful in the evaluation of their influence on the course of the diseases [46]. The only known simultaneous assessment of anti-ETAR and anti-CXCR3 antibodies is described in patients with Sjogren syndrome. In this case, the anti-CXCR3 antibody levels were lower, and anti-ETAR antibody levels were higher, than those of the healthy control group. Nevertheless, both these antibodies were changed simultaneously in this group of patients [34].

### 4.1. Study Limitations

- Our results require confirmation in bigger groups of patients.
- The molecular activity of anti-ETAR and anti-CXCR3 antibodies in specific glomerular diseases should be evaluated with molecular models.
- The influence of treatment on the course of the disease in prospective observations must be accounted for.

We suggest performing a meta-analysis of different studies and models using artificial intelligence to gain a more detailed view of the connections between different markers and antibodies in glomerular and other diseases. 

### 4.2. Future Perspectives

Anti-ETAR and anti-CXCR3 antibodies may be useful markers of IgA nephropathy and lupus nephritis prognosis. Anti-ETAR antibodies appear to be connected with the prognosis of membranous nephropathy, and anti-CXCR3 antibodies may also be a marker of the course of FSGS. The test for antibodies is relatively easy to perform, requiring only serum, and it may provide some important information. This may facilitate therapeutic decisions. Such tests may diminish the future necessity of kidney biopsies, avoiding the risk of complications that are connected with invasive procedures.

The potential influence of antibodies on the course of various diseases provides the opportunity to create specific molecular drugs influencing the course of a disease. It is possible to create monoclonal antibodies against antibodies or vaccines against receptors [47] and, in this way, influence the levels of anti-ETAR, anti-CXCR3, and other antibodies. There are some data indicating that everolimus may have an influence on the frequency of anti-ETAR antibodies’ appearance after kidney transplantation [48]. Anti-CXCR3 antibodies are postulated to be potential new therapeutic targets in cardioprotection [12], a very important topic for society, which increases the probability of creating new drugs acting on this axis in the future. A known CXCR3 antagonist, (±)-NBI-74330, has been found to promote beneficial effects in the control of pain in rat models [49]. When established in other areas, such drugs could be of interest to nephrologists to influence glomerular diseases.

We are the first to assess the anti-ETAR and anti-CXCR3 antibody levels in this set of different types of glomerular diseases.

## 5. Conclusions

Our results indicate lower anti-ETAR antibody levels in patients with FSGS and IgA nephropathy compared to the control group. Both types of antibodies correlated with creatinine levels after 2 years of observation in IgA nephropathy. Both types of antibodies seem to have a negative influence on total protein and albumin levels in systemic lupus erythematosus. 

This prospective observation showed that anti-ETAR and anti-CXCR 3 antibody levels are connected with the course of IgA nephropathy and lupus nephritis. An influence of ETAR antibodies on the course of membranous nephropathy, and CXCR3 antibodies on the course of FSGS, is also probable.

## 6. Disclosures

Biochemical blood analyses were performed by CELLTREND (Luckenwalde, Germany) free of charge.

Szymczak Maciej, Żabińska Marcelina, Janek Łucja, Wronecki Jakub, Kujawa Krzysztof, Kościelska-Kasprzak Katarzyna, Gołębiowski Tomasz, Banasik Mirosław, and Schulze-Forster Kai have no conflicts of interest to disclose. Harald Heidecke is the CEO and stakeholder of CellTrend GmbH.

## Figures and Tables

**Table 1:** Clinical data of patients with specific glomerular diseases (median values).

Disease	Creatinine (Serum) (mg/dL)	Proteinuria (g/24 h)	Total Protein (Serum) (g/dL)	Albumin (Serum) (g/dL)	Age (Years)	Sex (Percent of Males)
**membranous nephropathy (n = 18)**	1.25 (Range: 0.8–3.3)	2.64 (Range: 0.1–15.8)	4.8 (Range: 3.7–5.9)	2.8 (Range: 1.7–3.9)	51.5 (Range: 28–69)	55%
**focal and segmental glomerulosclerosis (n = 25)**	1.21 (Range: 0.73–3.19)	2.3 (Range: 0.07–13.99)	5 (Range: 3–7.3)	2.9 (Range: 1.3–4.8)	48 (Range: 19–74)	56%
**lupus nephritis (n = 17)**	1.06 (Range: 0.77–2.19)	1.59 (Range: 0.18–5.95)	5.5 (Range: 3.8–7.3)	3.1 (Range: 2–4)	34 (Range: 19–66)	47%
**IgA nephropathy (n = 14)**	1.06 (Range: 0.71–1.82)	0.94 (Range: 0.09–4.54)	5.65 (Range: 4.4–6.5)	3.4 (Range: 2.2–4)	45.5(Range: 20–60)	50%
**mesangial proliferative (non-IgA) glomerulonephritis (n = 6)**	0.93 (Range: 0.59–1.55)	2.58 (Range: 0.62–7.13)	4.8 (Range: 3.9–5.2)	2.8 (Range: 1.6–3.2)	28 (Range: 20–52)	50%
**control group (n = 22)**	1.2 (Range:0.9–1.3)	0 (Range:0–0)	7.4 (Range: 6.6–8.2)	4.4 (Range: 3.5–5.2)	44 (Range: 26–80)	50%
**c-ANCA vasculitis (n = 40)**	1.81 (Range: 0.69–7.78)	0.64 (Range: 0.06–19)	6.3 (Range: 5.3–7.1)	3.6 (Range: 2.4–4.6)	58 (Range: 21–81)	45%
p-ANCA vasculitis (n = 16)	3.13 (Range: 0.79–9.04)	1.73 (Range: 0.14–12.3)	5.95 (Range: 4.8–8.3)	3.5 (Range: 2.8–4.3)	62 (Range: 37–87)	56%

## Data Availability

The original contributions presented in this study are included in the article/Supplementary Materials. Further inquiries can be directed to the corresponding author.

## Supplementary Materials

The following supporting information can be downloaded at: https://www.mdpi.com/article/10.3390/jcm13247752/s1.

## Abbreviations

ACE	angiotensin II converting enzyme
ANA	antinuclear antibodies
ANOVA	analysis of variance
AT1R	angiotensin II type 1 receptor
cANCA	anti-neutrophil cytoplasmic antibodies
CD	cluster of differentiation
cGAMP	cyclic guanosine monophosphate–adenosine monophosphate
CXCR3	C-X-C motif chemokine receptor 3
ETAR	endothelin A receptor
FSGS	focal and segmental glomerulosclerosis
HLA	human leukocyte antigens
IgA	immunoglobulin A
IgG	immunoglobulin G
MHC	major histocompatibility complex
MPO	ANCA-myeloperoxidase anti-neutrophil cytoplasmic antibodies
pANCA	perinuclear anti-neutrophil cytoplasmic antibodies
POD	horseradish peroxidase
