# Supplementary figures and images for: The Influence of Anti-ETAR and Anti-CXCR3 Antibody Levels on the Course of Specific Glomerulonephritis Types
Source: J Clin Med. 2024 Dec 19;13(24):7752. doi: 10.3390/jcm13247752 (PMC11679591; doi:10.3390/jcm13247752)

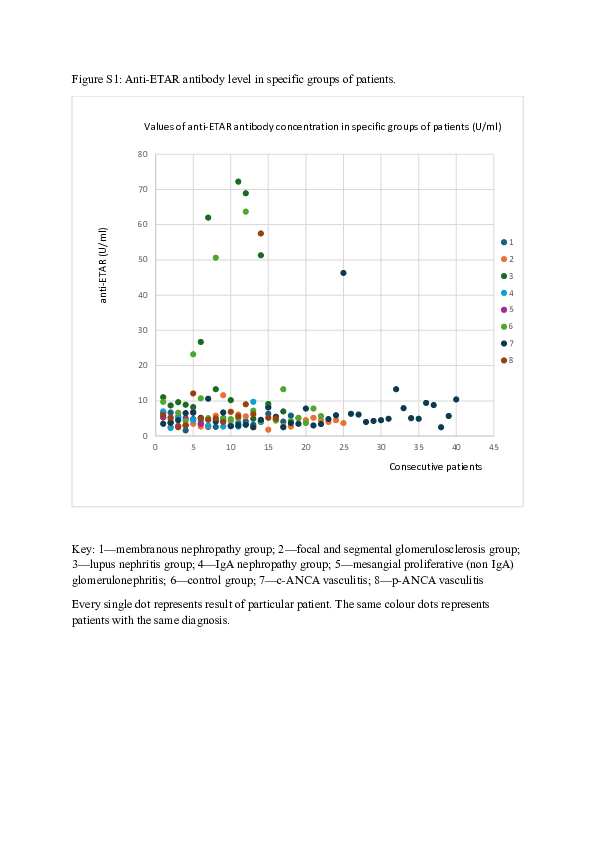

Supplement: Supplementary file 1 [file jcm-13-07752-s001.zip › Figure S1.jpg]

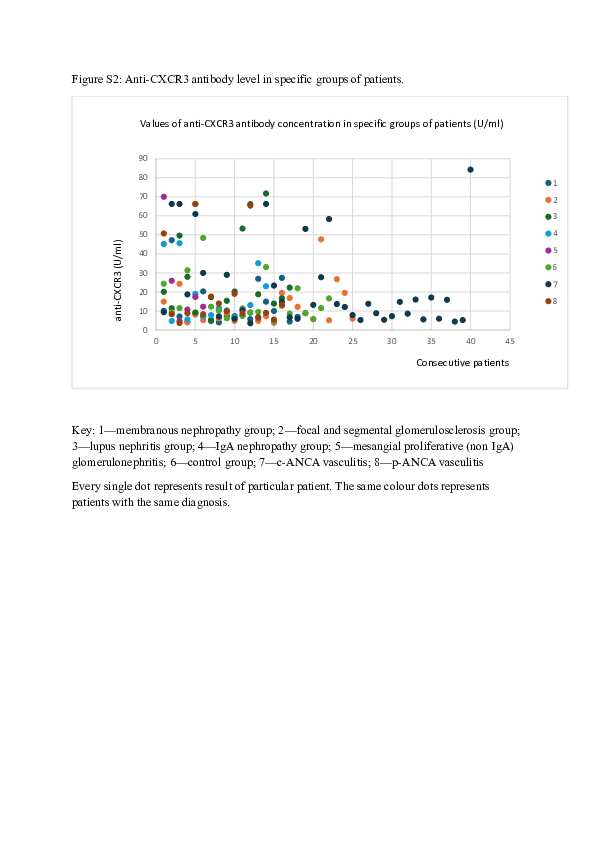

Supplement: Supplementary file 1 [file jcm-13-07752-s001.zip › Figure S2.jpg]
